# Modulating Surface Morphology Related to Crystallization Speed of Perovskite Grain and Semiconductor Properties of Optical Absorber Layer under Controlled Doping of Potassium Ions for Solar Cells

**DOI:** 10.3390/ma11091605

**Published:** 2018-09-04

**Authors:** Tao Ling, Xiaoping Zou, Jin Cheng, Ying Yang, Haiyan Ren, Dan Chen

**Affiliations:** Research Center for Sensor Technology, Beijing Key Laboratory for Sensor, Ministry of Education Key Laboratory for Modern Measurement and Control Technology, School of Applied Sciences, Beijing Information Science and Technology University, Jianxiangqiao Campus, Beijing 100101, China; taoling8102@gmail.com (T.L.); chengjin@bistu.edu.cn (J.C.); yang09960@gmail.com (Y.Y.); yanh3100@gmail.com (H.R.); danchen630@gmail.com (D.C.)

**Keywords:** solar cell, doping of K^+^, surface morphology, perovskite thin film, crystallization speed, semiconductor properties

## Abstract

Perovskite thin films with excellent optical semiconductor and crystallization properties and superior surface morphology are normally considered to be vital to perovskite solar cells (PSCs). In this paper, we systematically survey the process of modulating surface morphology and optical semiconductor and crystallization properties of methylammonium lead iodide film by controlling doping of K^+^ for PSC prepared in air and propose the mechanism of large K^+^-doped perovskite grain formation related to crystallization speed. The increase in the crystallization speed leads to the production of large grains without localized-solvent-vapor (LSV) pores via moderate doping of K^+^, and the exorbitant crystallization speed induces super large grains with LSV pores via excessive doping of K^+^. Furthermore, the semiconductor properties (absorption band edge wavelength, PL emission peak wavelength, energy band gap) of perovskite film can be significantly tuned by controlled doping of K^+^. The investigation of the detailed process of modulating surface morphology and semiconductor properties of perovskite thin film by controlled doping of K^+^ may provide guidance and pave the way for superior component design of absorption materials for cost-efficient PSCs.

## 1. Introduction

Recently, perovskite solar cells (PSCs) were considered, by the public, as the most promising alternative photovoltaic devices as a result of their simple process and high efficiency [1,2,3,4,5]. Undoubtedly, perovskite thin film with superior surface morphology (such as better flatness, low defect density, and large and dense crystal grains) [6,7,8,9] and excellent semiconductor properties (such as suitable exciton binding energy, long carrier lifetime, and appropriate band gap) [6,10,11,12], is normally considered to be vital for PSCs. In order to optimize some semiconductor properties of perovskite materials (such as the interface energy barrier, contact resistance, and carrier diffusion length), some intended doping has been used in absorbing materials based on photovoltaic devices [6,13,14,15,16,17,18,19,20,21,22].

Previously, there were some published reports, which showed that Cs- and Rb-doped PSCs had better stability and higher power conversion efficiency (PCE) than pure PSCs [3,15,16,17]. In addition, some research groups reported that K- and Na-doped perovskites also had better PCE [5,18,19,20,21,22]. Huang et al. confirmed that Na^+^ can decrease defect density in perovskite absorbers [22]. In our research group, Bai and Yang demonstrated that Rb^+^ [11] and Na^+^ [12] can change carrier concentration and mobility, respectively, in perovskite absorbers.

Specially, Tang et al. found that K^+^ can eliminate the barrier and reduce the defect of perovskite [5,23]. In addition, Zhao et al. reported the modified surface work function, improved crystallinity, and prolonged carrier lifetime in perovskite film via doping of K^+^ [20]. Yao et al. demonstrated that K^+^ prefers to occupy the interstitial site in the lattice of perovskite [24]. Kubicki et al. reported that there are unreacted KI and KPbI_3_ in K-doped perovskite thin film [25]. Abdi-Jalebi et al. reported the substantial mitigation of both non-radiative losses in perovskite films and interfaces with passivating potassium halide layers [26]. Although there are a few reports on doping of K^+^ in perovskite materials [5,18,20,23,24,25,26,27], a detailed report on the mechanism of large perovskite grain formation related to crystallization speed (CS) in K^+^-doped perovskite thin films has not been seen up to now, which is also important for the research of K^+^-doped perovskite thin film for solar cells.

Although certified PCE > 20% was obtained via tuning of the process and composition of perovskite thin film of PSCs [28,29], these devices have been obtained by various processes, which contains a necessary step of forming counter electrode (CE) by thermal evaporation of metals. Apparently, the costs of these metallic CEs are relatively high. To reduce the costs of PSCs, some researchers have developed carbon CEs [30,31,32]. Yang et al. prepared a 2.6% efficient original perovskite solar cell with a candle soot carbon/FTO CE [30]. In our research group, previously, a spongy carbon/FTO composite structure was adopted as a CE and the corresponding cell achieved 4.24% PCE [33], and recently, a PSC with this kind of composite CE was prepared via sequential deposition route and achieved 10.7% PCE [34].

This time, we systematically survey the process of modulating surface morphology and optical semiconductor and crystallization properties of methylammonium lead iodide (MAPbI_3_) thin film via controlled doping of K^+^ for PSC prepared in air. In addition, we propose the mechanism of large perovskite grain formation on CS and doping concentration of K^+^. The increase in the CS leads to the production of large grains without localized-solvent-vapor (LSV) pores via moderate doping of K^+^ and the exorbitant crystallization speed induces super large grains with LSV pores via excessive doping of K^+^. Furthermore, the optical semiconductor and crystallization properties of perovskite film prepared in air could be significantly tuned by doping of K^+^. We also observed the transition from blue shift to red shift of the absorption band edge wavelength with the increase in the amount of doping of K^+^, which is consistent with the shift of the PL emission peak wavelength. Although this transition did not exist in the results of most research groups [5,20,23], it has occurred [18]. The PSC was prepared via a one-step solvent process under ambient conditions and also resulted in a promising 8.14% PCE on the area of 0.2 cm^2^.

## 2. Materials and Methods

### 2.1. Materials

Fluorine-doped SnO_2_ (FTO) substrates were purchased from Dalian HeptaChroma Solar Technology Development Corp (Dalian, China). Dimethyl sulfoxide (DMSO) and N,N-dimethylformamide (DMF) were obtained from Sa’en Chemical Technology Corp (Shanghai, China). 18NR-T TiO_2_ paste (M-TiO_2_), isopropyl alcohol (IPA), PbI_2_, chlorobenzene, and acidic titanium dioxide solution (C-TiO_2_) were obtained from Shanghai MaterWin New Materials Corp (Shanghai, China). Spiro-OMeTAD solution, Methylammonium iodide (MAI), and KI were obtained from Xi’an Polymer Light Technology Corp (Xi’an, China).

### 2.2. Device Fabrication

The cleaning method of FTO and deposition processes of C-TiO_2_ layer, M-TiO_2_ layer and Spiro-OMeTAD layer are the same as those previously [34]. The preparation method of the perovskite thin film deposit and counter electrode will be introduced next. MAPbI_3_ solution was prepared by mixing 1.2 M CH_3_NH_3_I and 1.3 M PbI_2_ in a mixed solvent (DMF:DMSO = 4:1). Then, 0.6, 0.9 and 1.2 M KI solution was prepared by dissolving a corresponding amount of KI solid in 200 μL mixed solvent (DMF:DMSO = 4:1). These solutions were added to 1 mL prepared MAPbI_3_ solution. These precursor solutions were spin coated on the M-TiO_2_/C-TiO_2_/FTO substrate through two periods at 1000 rpm for 10 and 3000 rpm for 30 s. During the second period, 20 μL of chlorobenzene was dropped at 15 s prior to the end of the period. Then, a thermal annealing of 100 °C for 15 min was processed to yield perovskite films. Finally, the FTO substrates were used to collect the soot of a burning candle as spongy carbon CEs and these spongy carbon films on FTO glasses were then pressed on the as-prepared uncompleted devices.

### 2.3. Characterization

The details of scanning electron microscopy (SEM) with energy spectrum analysis, X-ray diffraction (XRD) are the same as those used before [34]; however, the absorption spectra of perovskite films with different doping concentrations of K^+^ were analyzed by an ultraviolet visible (U-V) absorption spectrometer (Avantes, Apeldoom, The Netherlands) and the PL was examined by a LabRAW HR800 PL testing system (HORIBA JObin Yvon, Paris, France).

## 3. Results and Discussion

**Figure 1:**
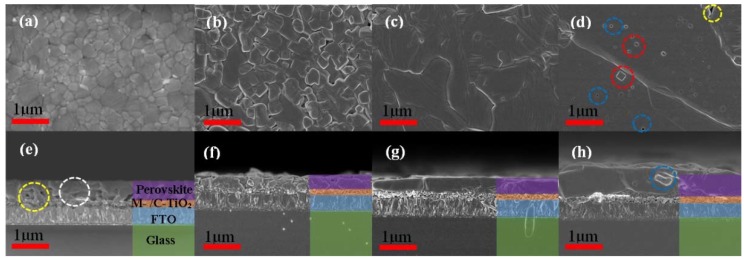
Surface and cross-sectional SEM images for (**a**,**e**) MAPbI_3_+0M; (**b**,**f**) MAPbI_3_+0.6M; (**c**,**g**) MAPbI_3_+0.9M; (**d**,**h**) MAPbI_3_+1.2M.

The K^+^ doping contents of 1.2 mL perovskite precursor solutions with the addition of 200 μL KI solutions of 0 M, 0.6 M, 0.9 M and 1.2 M were named 0M, 0.6M, 0.9M and 1.2M, respectively. The MAPbI_3_ films with 0 M, 0.6 M, 0.9 M and 1.2 M were named MAPbI_3_+0M, MAPbI_3_+0.6M, MAPbI_3_+0.9M and MAPbI_3_+1.2M, respectively. Surface and cross-sectional SEM images for perovskite absorber layers with 0 M, 0.6 M, 0.9 M and 1.2 M are presented in Figure 1, where we can see clearly the process of modulating surface morphology of the perovskite thin film via controlled doping of K^+^. In Figure 1e–h, we can see clearly the perovskite layer, M-/C-TiO_2_ (Mesoporous-TiO_2_ and Compact-TiO_2_) layer and FTO layer from the top to the bottom. Although the surface of MAPbI_3_+0M (presented in Figure 1a) is relatively flat, there are many pores (presented in yellow circle, Figure 1e) in the interface between MAPbI_3_+0M and M-/C-TiO_2_. In addition, the sizes of grains are relatively smaller and there are more vertical grain boundaries in MAPbI_3_+0M (presented in the white circle, Figure 1e). Relative to MAPbI_3_+0M, the surface of MAPbI_3_+0.6M (presented in Figure 1b) is rough, but pores are not present in the interface between perovskite layer and M-/C-TiO_2_, which means the doping of K^+^ can passivate the interface between MAPbI_3_+0M and M-/C-TiO_2_. Furthermore, the sizes of grains are bigger, and there are fewer transverse grain boundaries (presented in Figure 1b). As for MAPbI_3_+0.9M (Figure 1c,g), the surface has better flatness and grains are larger than for MAPbI_3_+0.6M, so that the transverse grain boundaries are greatly reduced and vertical grain boundaries are almost gone, which lead to a great improvement in its efficiency. From Figure 1d, we can find a super large grain (>4 μm in the longest direction), but there are LSV pores (presented in blue circle), boundary gaps (presented in yellow circle) and unidentified square protrusions (presented in red circle) on the surface of MAPbI_3_+1.2M. It is more important to note that there are also LSV pores (presented in blue circle) inside MAPbI_3_+1.2M according to Figure 1h, which greatly reduces the efficiency of the device. These LSV pores are caused by the inefficient discharge of the solvent from the interior of crystallizing perovskite due to the exorbitant perovskite CS. Through the comparison of these cross-sectional SEM images (Figure 1e–h), we can find that MAPbI_3_+1.2M becomes very thick (~900 nm). Furthermore, the perovskite grain sizes increases (~500 nm, ~700 nm, ~2 μm and ~4 μm, on average, corresponding MAPbI_3_+0M, MAPbI_3_+0.6M, MAPbI_3_+0.9M and MAPbI_3_+1.2M, respectively) with the increase in the doping concentration of K^+^ according to the comparison of these surface SEM images (Figure 1a–d). The perovskite grain sizes increase with the increase in the doping concentration of K^+^ so that the number of grain boundaries of perovskite films decreases. In addition, the decomposition of perovskite films begins at the boundaries of perovskite grains, so the stability of devices increases with the increase in the doping concentration of K^+^.

**Figure 2:**
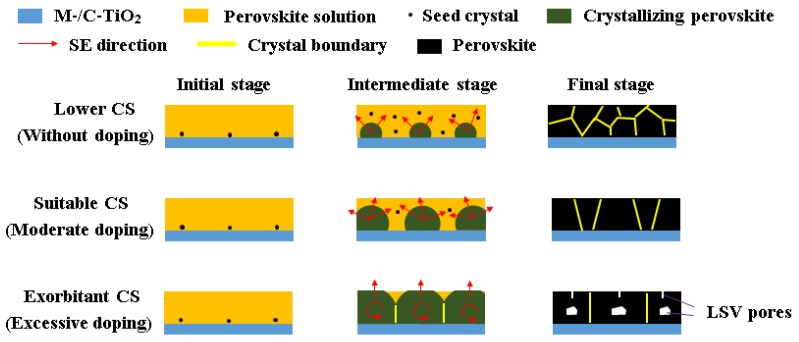
Schematic illustration for the mechanism of large K^+^-doped perovskite grain formation related to crystallization speed.

According to these experimental results, we propose the following mechanism (presented in Figure 2) of large K^+^-doped perovskite grain formation related to CS. In Figure 2, the red arrow represents the solvent evaporation (SE) direction and the small black spot represents seed crystal, which is the root of the formation of perovskite grain. We assume that the distributions of seed crystals in all samples are the same during the initial stage. Lower CS (corresponding to the absence of doping) leads to relatively more spaces to form seed crystals in the perovskite solution due to smaller crystallizing grains during the intermediate stage, which results in smaller grains and more grain boundaries during the final stage eventually. Suitable CS causes larger crystallizing perovskite grains and less spaces for the formations of new seed crystals during the intermediate stage via moderate doping of K^+^, and finally, the large grains without LSV pores formed. However, excessive doping can cause exorbitant CS and leads to super large crystallizing grain during the intermediate stage, so that there are almost no spaces to form new seed crystals and the solvent can only be evaporated upwards for filling of the whole space by the transverse crystallizing grains, which cause the solvent to not effectively discharge from the interior of crystallizing perovskite; as a result, LSV pores are formed inside the perovskite thin films and on its surface during the final stage. The crystallization already starts when the seed crystal form and it may be during solvent evaporation when the spin-coating or the process of chlorobenzene addition or thermal annealing occur. Irrespective, the schematic mechanism in Figure 2 is adaptive. In addition, it is worth emphasizing that new seed crystals may form at all times, and once the seed crystals form, the process described by the schematic mechanism takes place.

**Figure 3:**
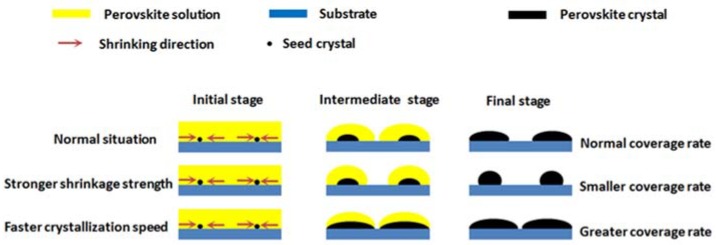
Schematic illustration for the formation of different coverage rates on the hydrophobic substrate due to different solution thin film shrinkage strength and the crystallization speed of perovskite.

To back up this theory that the CS of perovskite increases with the increase in the K doping amount, we analyze the results of previous research. Uz Zaman et al. prepared K-doped perovskite thin films on FTO substrates by spin coating [35], which are hydrophobic substrates, and the perovskite solution films must shrink after spin coating, unavoidably so that the final perovskite films cannot completely cover the substrates. We can find that the coverage area of perovskite thin films increases with the increase of the K doping amount according to their SEM results [35]. The coverage rate of perovskite thin film on substrate is mainly determined by the strength of the solution thin film shrinkage and the CS of perovskite, illustrated in Figure 3. In Figure 3, the red arrow marks the shrinkage direction of the perovskite solution thin film and the small black dot represents the perovskite seed crystal. In the process of perovskite solution film shrinking, perovskite crystals are also growing in areas with perovskite precursor solution, so that stronger shrinkage strength leads to a smaller coverage rate and faster crystallization speed leads to a greater coverage rate. The shrinkage strength of the perovskite solution film is mainly determined by the solvent and substrate; however, the solvents of the perovskite precursor solutions with different K content and substrates are exactly the same, so the shrinkage strengths of perovskite solution films are almost the same. Thus, the conclusion can be reached that different crystallization speeds lead to different perovskite film coverage rates, and the crystallization speed of perovskite increases with the increase in the K doping amount. The crystallization speed of perovskite we expect can make the grain grow to a large state without localized-solvent-vapor (LSV) pores or with small number of LSV pores of which the inducing efficiency attenuation is less than the benefit from the increase in grain size. According to SEM diagrams and JV curves, the expected speed is between the speed corresponding 0.9 M and the speed corresponding 1.2 M because MAPbI_3_+0.9M does not have LSV pores and MAPbI_3_+0.9M has LSV pores, which induce efficiency attenuation. Exorbitant crystallization speed can make the grain grow to a large state with more LSV pores of which the inducing efficiency attenuation is higher than the benefit from the increase in grain size, so the speed corresponding to 1.2 M is an exorbitant crystallization speed.

**Figure 4:**
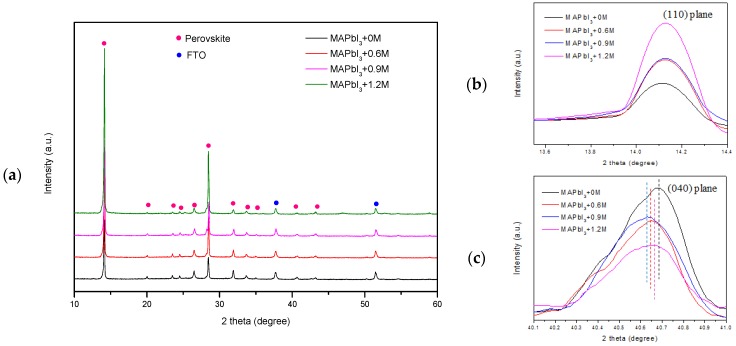
XRD patterns of perovskite films with 0 M, 0.6 M, 0.9 M and 1.2 M: (**a**) whole patterns; (**b**) enlargement of (110) crystal plane; (**c**) enlargement of (040) plane.

Figure 4a shows XRD pattern of perovskite thin films with different doping concentration of K^+^ and we can find that the peak position of perovskite crystal has not been transferred, which means there is no change in the type of perovskite crystal structure via different concentration doping of K^+^ [36]. In addition, the perovskite film exhibits pure tetragonal phase according to Figure 4a, which indicates that the K^+^ enters into the perovskite lattice successfully [20]. From Figure 4b, we noticed that the perovskite crystallization is improved with the increase in the K doping amount, which indicates a longer carrier lifetime [20]. Compared to MAPbI_3_+0M, almost all other (040) peaks shift to lower degree in Figure 4c, which means K^+^ mainly occupied interstitial sites in lattices [20].

**Figure 5:**
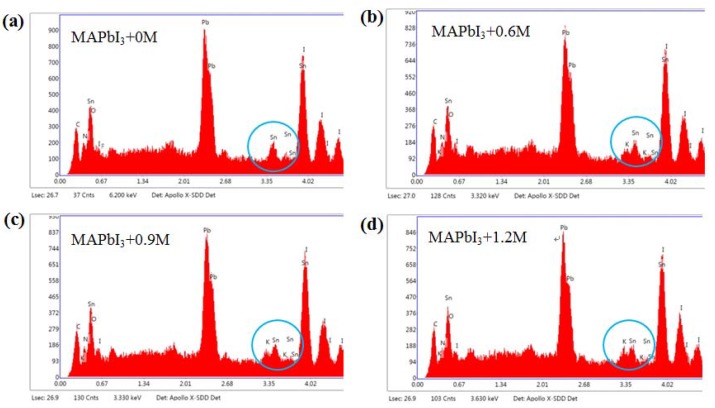
The Energy dispersive X-ray Spectroscopy (EDX) of perovskite films with 0 M, 0.6 M, 0.9 M and 1.2 M.

The Energy dispersive X-ray Spectroscopy (EDX) of K-doped perovskite crystallite films and the specific values of quantifying doping levels are presented in Figure 5 and Table 1, respectively. In the EDXs (Figure 5a–d), we can see that the peaks of K elements (presented in blue circle) increase with the increase in the doping concentration, which is consistent with the values in Table 1. The excess iodine coming from the KI solution can also affect the perovskite film performance, which is carried out by Equation (1) [37] and Equation (2) [37,38].

2 I^−^ → I_2_ + 2 e^−^ (1)

I^−^ + I_2_ → I_3_^−^ (2)

However, I_3_^−^ can effectively decrease the concentration of defects in organic-inorganic lead halide perovskite films [38]. 

**Figure 6:**
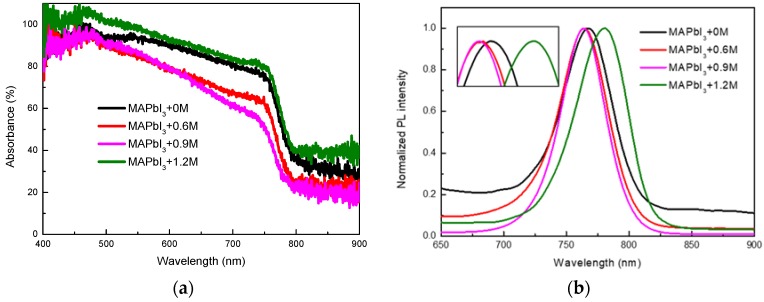
(**a**) Absorbance spectra and (**b**) PL spectra of perovskite thin films with 0 M, 0.6 M, 0.9 M and 1.2 M.

Figure 6a,b show the absorbance spectra and PL spectra of perovskite thin films with 0 M, 0.6 M, 0.9 M and 1.2 M, respectively. There are blue shifts of the absorption band edge wavelength and PL emission peak wavelength of MAPbI_3_+0.6M and MAPbI_3_+0.9M, and the red shift the absorption band edge wavelength and PL emission peak wavelength of MAPbI_3_+1.2M has taken place, compared to reference MAPbI_3_+0M, indicating that the energy band gap increases first and then decreases with the increase in the doping concentration of K^+^ [5].

**Figure 7:**
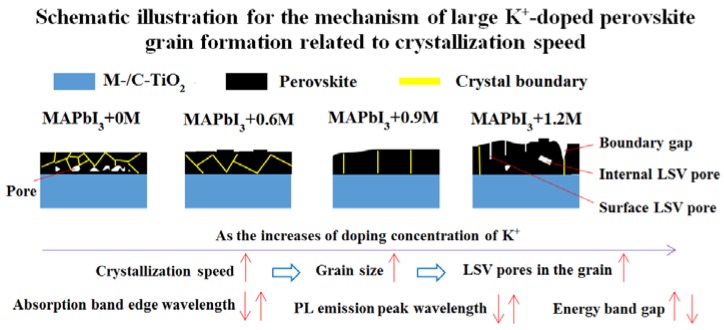
Schematic illustration for the process of modulating surface morphology related to CS and optical semiconductor properties of perovskite thin film via controlled doping of K^+^ prepared in air.

Schematic illustration for the process of modulating surface morphology related to CS and optical semiconductor properties of perovskite thin films via controlled doping of K^+^ is shown in Figure 7. It gives a very detailed description of the process of modulating surface morphology (including grain size, surface flatness, transverse and vertical grain boundary quantity, internal LSV pores, surface LSV pores, pores in the interface between perovskite layer and M-/C-TiO_2_, thickness of perovskite thin film, boundary gap) and optical semiconductor properties (absorption band edge wavelength, PL emission peak wavelength, energy band gap) of perovskite thin film via controlled doping of K^+^ prepared in air. It is worth emphasizing that the mechanism of large K^+^-doped perovskite grain formation is related to CS. The doping of K^+^ leads to an increase in CS of perovskite grains during the intermediate stage, and results the formation of large perovskite grains during the final stage. However, excessive doping of K leads to a rapid perovskite crystallization speed that induces super large crystallizing grains and causes the solvent to not effectively discharge from crystallizing perovskite during the intermediate stage; as a result, LSV pores are formed inside the perovskite thin films and on its surface during the final stage.

**Figure 8:**
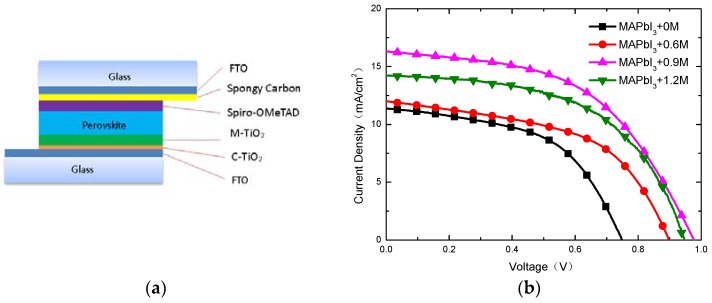
(**a**) Schematic illustration for the structure of devices; (**b**) Light current-voltage (J-V) curves from reverse scan (RS) for the devices with 0 M, 0.6 M, 0.9 M and 1.2 M under RS.

The structure of the device is shown in Figure 8a. Figure 8b shows the J-V curves from RS for the devices with different doping concentrations of K^+^, measured under simulated sunlight (AM 1.5 G), and the detail photovoltaic parameters of PSCs is shown in the Table 2. V_oc_ has increased significantly via controlling doping of K^+^, as a result of elimination of pores in the interface between the perovskite layer and M-/C-TiO_2_ layer. Due to larger grains in the MAPbI_3_+0.9M and MAPbI_3_+1.2M, the corresponding devices obtain higher J_sc_. Although the absorption spectrum of MAPbI_3_+0.9M is relatively low compared to other perovskite films, its device yielded the best performance (especially the V_oc_ and J_sc_) due to prolonged carrier lifetime, improved surface morphology and crystallinity, which means the photovoltaic properties of the devices are improved via appropriate doping of K^+^. A promising efficiency of 8.14% was achieved for the 0.9 M K^+^-doped device with the carbon/FTO CE, and these simple and low-cost preparation techniques of high-class perovskite thin films and carbon/FTO CE under ambient conditions are beneficial for promoting commercialization.

## 4. Conclusions

The LSV-pore-free perovskite grains became significantly larger via moderate doping of K^+^. The mechanism of large K^+^-doped perovskite grain formation is related to CS and the theory that the CS of perovskite increases with the increase of K doping amount was suggested and preliminarily confirmed. The detailed description of the process of modulating surface morphology (including grain size, surface flatness, transverse and vertical grain boundary quantity, internal LSV pores, surface LSV pores, pores in the interface between MAPbI_3_+0M and M-/C-TiO_2_, thickness of perovskite thin film, boundary gap) and optical semiconductor properties (absorption band edge wavelength, PL emission peak wavelength, energy band gap) of perovskite thin film via controlled doping of K^+^ prepared in air was presented. A promising efficiency of 8.14% in a size of 0.2 cm^2^ was achieved for the device with inexpensive carbon/FTO CE under one sun illumination. Obviously, mass production of cost-efficient K^+^-doped PSCs, with spongy carbon/FTO CEs, under ambient atmosphere, is possible.

## Figures and Tables

**Table 1:** EDX identified element percentages (weight %) in the perovskite films with 0 M, 0.6 M, 0.9 M and 1.2 M.

Sample	K K	C K	N K	O K	Pb M	Sn L	I L
MAPbI3+0M	0	5.10	2.35	0.80	23.49	5.84	62.42
MAPbI3+0.6M	1.44	4.58	2.18	0.49	21.77	5.40	64.14
MAPbI3+0.9M	1.50	4.59	1.73	0.38	22.21	5.67	63.92
MAPbI3+1.2M	1.80	4.50	2.07	0.55	21.71	5.26	64.11

**Table 2:** Photovoltaic parameters of PSCs with 0 M, 0.6 M, 0.9 M and 1.2 M under RS.

DC ^a^	J_sc_^b^ (mA/cm^−2^)	V_oc_^c^ (v)	FF ^d^	PCE (%)
0 M	11.37	0.75	0.52	4.43
0.6 M	12.01	0.90	0.52	5.62
0.9 M	16.33	0.98	0.51	8.14
1.2 M	14.20	0.95	0.55	7.42

^a^ DC: Doping concentration of K^+^; ^b^ J_sc_: Short-circuit photocurrent density; ^c^ V_oc_: Open-circuit voltage; ^d^ FF: Fill factor.
